# Accuracy of MR neurography as a diagnostic tool in detecting injuries to the lingual and inferior alveolar nerve in patients with iatrogenic post-traumatic trigeminal neuropathy

**DOI:** 10.1007/s00330-023-10363-2

**Published:** 2023-12-04

**Authors:** Mado Bangia, Iraj Ahmadzai, Jan Casselman, Constantinus Politis, Reinhilde Jacobs, Fréderic Van der Cruyssen

**Affiliations:** 1 Department of Oral & Maxillofacial Surgery, University Hospitals Leuven, Kapucijnenvoer 33, 3000 Louvain, Belgium; 2 Department of Imaging and Pathology, OMFS-IMPATH Research Group, Faculty of Medicine, University Leuven, Louvain, Belgium; 3 Department of Radiology, AZ St-Jan Brugge-Oostende, Brugge, Belgium; 4 Department of Radiology, AZ St-Augustinus, Antwerp, Belgium; 5 University Ghent, Ghent, Belgium; 6 Dentomaxillofacial Imaging Center, University Hospitals Leuven, Louvain, Belgium; 7 Department of Dental Medicine, Karolinska Institutet, Stockholm, Sweden

**Keywords:** Trigeminal neuropathy, Trigeminal nerve injuries, Magnetic resonance imaging

## Abstract

**Objectives:**

MR neurography has the ability to detect and depict peripheral nerve injuries. This study evaluated the potential of MR neurography in the diagnosis of post-traumatic trigeminal neuropathy.

**Methods:**

Forty-one participants prospectively underwent MR neurography of the lingual and inferior alveolar nerves using a 3D TSE STIR black-blood sequence. Two blinded and independent observers recorded the following information for each nerve of interest: presence of injury, nerve thickness, nerve signal intensity, MR neurography Sunderland class, and signal gap. Afterwards, the apparent nerve-muscle contrast-to-noise ratio and apparent signal-to-noise ratio were calculated. Clinical data (neurosensory testing score and clinical Sunderland class) was extracted retrospectively from the medical records of patients diagnosed with post-traumatic trigeminal neuropathy.

**Results:**

Compared to neurosensory testing, MR neurography had a sensitivity of 38.2% and specificity of 93.5% detecting nerve injuries. When differentiated according to clinical Sunderland class, sensitivity was 19.1% in the presence of a low class injury (I to III) and improved to 83.3% in the presence of a high class (IV to V). Specificity remained unchanged. The area under the curve using the apparent nerve-muscle contrast-to-noise ratio, apparent signal-to-noise ratio, and nerve thickness to predict the presence of an injury was 0.78 (*p* < .05). Signal intensities and nerve diameter increased in injured nerves (*p* < .05). Clinical and MR neurography Sunderland scores positively correlated (correlation coefficient = 0.53; *p* = .005).

**Conclusions:**

This study shows that MR neurography can accurately differentiate between injured and healthy nerves, especially in the presence of a more severe nerve injury.

**Clinical relevance statement:**

MR neurography is not only able to detect trigeminal nerve injuries, but it can also provide information about the anatomical specifications of the injury, which is not possible with clinical neurosensory testing. This makes MR neurography an added value in the management of post-traumatic trigeminal neuropathy.

**Key Points:**

*• The current diagnosis of post-traumatic trigeminal neuropathy is mainly based on clinical examination.*

*• MR neurography is able to visualize and stratify peripheral trigeminal nerve injuries.*

*• MR neurography contributes to the diagnostic process as well as to further decision-making.*

## Introduction

The trigeminal nerve (TN) provides sensation to the face via its three major branches: the ophthalmic, maxillary, and mandibular nerves. The latter has an additional function in supplying innervation to the muscles responsible for biting and chewing. Maxillofacial surgery and dental procedures (e.g., implant placement, molar tooth extraction, local anesthesia) have a risk of damage to one of these branches, which can result in the development of neurosensory deficiencies [1–3], a condition called iatrogenic post-traumatic trigeminal neuropathy (PTN). When accompanied with pain, the term post-traumatic trigeminal neuropathic pain is used. The lingual nerve (LN) and inferior alveolar nerve (IAN) are most frequently affected [1, 3]. Post-traumatic trigeminal neuropathic pain is described by the International Classification of Orofacial Pain (ICOP) as “unilateral or bilateral facial or oral pain following and caused by trauma to the trigeminal nerve(s), with other symptoms and/or clinical signs of trigeminal nerve dysfunction, and persisting or recurring for more than three months [4].” It suggests the following criteria for diagnosis [4]: pain in an area innervated by the TN, association of this pain with signs of nerve dysfunction in that same area, history of an injury to the TN, onset of the pain within 6 months after the injury, not better accounted for by another ICOP or International Classification of Headache Disorders version 3 (ICHD-3) diagnosis, and the presence of a lesion to the TN, which should be able to explain the pain, confirmed by a diagnostic test. The same criteria can be used for PTN. In current clinical practice, the diagnosis of PTN is primarily based on the patient’s history, description of symptoms, and physical and neurological examinations. The diagnostic test of use is clinical neurosensory testing (NST) [5]. NST findings can be translated into a degree of injury similar to the Sunderland classification, which correlates with surgical findings [6]. The different Sunderland classes were designed to provide information regarding prognosis (e.g., the possibility of functional recovery) and whether surgical treatment is needed to functionally recover [7]. Despite having the advantage of being easily accessible and non-invasive, this diagnostic approach has the disadvantage of being subjective and difficult to standardize. Furthermore, this approach is not able to provide information about the location and other anatomical specifications of the injury, which can be important in surgical planning. An accurate diagnostic tool that, ideally, is able to provide additional information about location, anatomical specifications, and degree of injury is necessary to make the right diagnostic and therapeutic decisions. An imaging modality called MR neurography (MRN) was designed to adequately visualize peripheral nerves, such as the LN and IAN. It has shown potential in depicting and diagnosing, as well as stratifying, peripheral nerve injuries [8–10]. However, most of these studies have some shortcomings in their methodology [11]. We conducted a prospective, blinded, and standardized study about the potential of MRN in detecting injuries to the LN and IAN in patients with PTN. The secondary objectives were to demonstrate that MRN is able to stratify nerve injuries, elucidate how to differentiate injured and healthy nerves using MRN, and illustrate the potential of individual MRN parameters in predicting nerve injury.

## Methods

This study was performed at the Department of Oral and Maxillofacial Surgery at University Hospitals Leuven, Belgium. Ethical approval was received from the Ethics Committee of the University Hospital Leuven (S61077). Informed consent was obtained from all participants.

### Participant

A total of 30 patients referred with orofacial neuropathy upon their visit to the Department of Oral and Maxillofacial Surgery at University Hospitals Leuven between June 2020 and June 2021 were recruited for the present study. The case series consisted of patients who fulfilled the following criteria: diagnosis of PTN (with or without pain) based on the ICOP criteria, clinical evidence of involvement of the LN or IAN, and an iatrogenic traumatic cause of injury. Patients who did not meet the inclusion criteria served as the control group together with 11 healthy volunteers. Age and gender were recorded for all participants.

### Image acquisition and analysis

MRN examinations were prospectively acquired at the Radiology Department at University Hospitals Leuven on an Ingenia 3-Tesla MR scanner (Philips Medical Systems) using a 32-channel standard head coil. We used the 3D cranial nerve imaging sequence (3D CRANI), a newly developed 3D TSE STIR black-blood sequence [12, 13]. It uses a pseudo steady-state (PSS) sweep in combination with a motion-sensitized driven equilibrium (MSDE) pulse and is able to suppress signals from fat, muscle, and blood to generate a nerve-selective image [12].

Gadolinium contrast was administered. The same examination protocol was used in all participants.

**Fig. 1:**
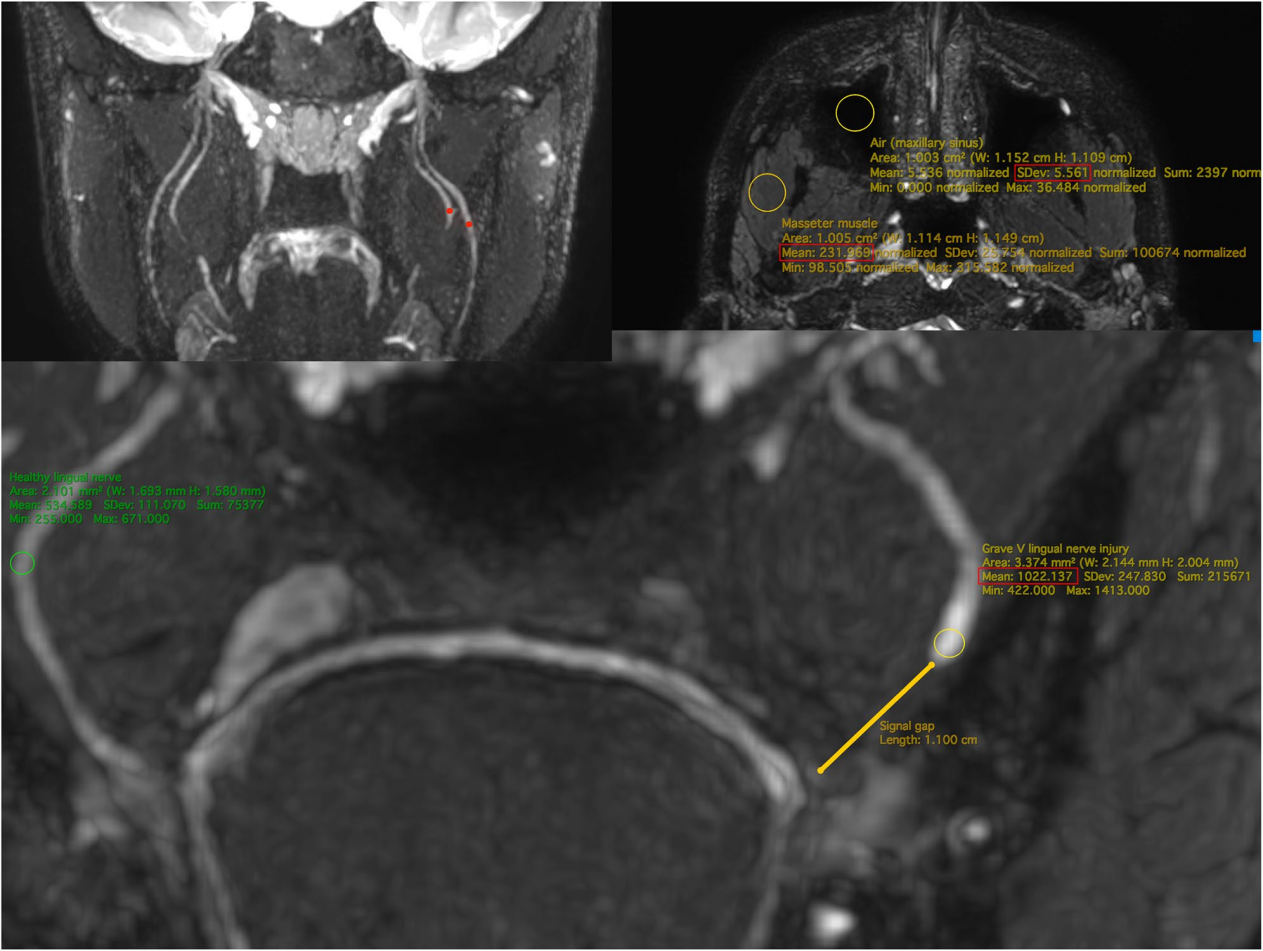
ROI placement, measurements, and calculations. Left upper panel: The coronal plane 3D CRANI image displays signal intensity measurements made by placing circular regions of interest (ROIs) within a healthy lingual nerve (LN) and inferior alveolar nerve (IAN) at predetermined landmarks: the mid-mandibular canal for the IAN and the point of maximum curvature for the LN. Right upper panel: Measurement of signal intensity within the masseter muscle (mROI) and within the air of the maxillary sinus (aROI). Lower panel: Left-sided grade V lingual nerve injury. The signal gap is measured between the proximal and distal nerve stumps. Next, a proximal ROI is determined within the nerve contours. On the right side, the signal intensity is measured at the predetermined landmarks. The apparent signal-to-noise ratio (aSNR) is measured using the formula iROI/SDair, and the apparent nerve-muscle contrast-to-noise ratio (aNMCNR) is calculated by (iROI − mROI)/SDair. Here is an example of an aSNR calculation for this grade V lingual nerve injury: aSNR = 1022.137/5.561 = 183.80. An example of an aNMCNR calculation for this grade V lingual nerve injury is as follows: aNMCNR = (1022.137 − 231.969) / 5.561 = 142.09

We recruited two independent junior researchers as observers to rate the images and extract information using a standardized questionnaire. They both received training on MRN interpretation and a calibration session was organized to familiarize themselves with MRN injury grading. They were blinded to the patient’s clinical history and diagnosis. Presence of injury (yes/no), nerve thickness (mm), and signal intensity of the nerve were to be determined for each nerve of interest (left LN, right LN, left IAN, and right IAN). Signal intensities were measured by placing circular regions of interest (ROIs) within the identified nerves (iROI) (Fig. 1). The same was done for the masseter muscle (mROI) and air (aROI) measured inside the maxillary sinus using circular ROIs of 1 cm^2^.

Measurements were made on axial/coronal reformatted images at predetermined standardized locations. If the site was injured, the measurement was made just proximal to the injury. If normal, the mid-mandibular canal (for the IAN) and maximum curvature of the LN were used as reference standards.

Furthermore, if an injury was thought to be present, the observers were asked to give that injury a score based on the MRN Sunderland classification criteria (Table 1) [8]. If it was not possible to assign a classification with confidence (e.g., an injury classified as III/IV), it was classified as indeterminate. If an injury was classified as class V, they were asked to measure the signal gap in millimeters.

**Table 1:** MRN Sunderland classification demonstrating the varying degrees of injury

Class	MRN
I	Qualitative: Homogeneously increased T2 signal for nerve with no change in caliber Quantitative: No changes
II	Qualitative: Homogeneously increased T2 signal for nerve and mild nerve thickening, perineural fibrosis Quantitative: < 50% larger than contralateral/normal nerve
III	Qualitative: Homogeneously increased T2 signal for nerve and moderate to marked nerve thickening, perineural fibrosis Quantitative: > 50% larger than contralateral/normal nerve
IV	Qualitative: Heterogeneously increased T2 signal for nerve and focal enlargement in otherwise continuous nerve (neuroma in continuity), perineural and intraneural fibrosis Quantitative: Focal swelling with heterogeneous T2 signal or fascicular disruption
V	Qualitative: Discontinuous nerve with end-bulb neuroma Quantitative: Complete disruption with gap and end-bulb neuroma

*MRN* MR neurography

All measurements were recorded in a spreadsheet for data analysis. Afterwards, the apparent nerve-muscle contrast-to-noise ratio (aNMCNR) and apparent signal-to-noise ratio (aSNR) were calculated for each nerve using the following formulas [13, 14]: iROI ÷ SD_air_ and iROI − mROI ÷ SD_air_.

Nerves that could not be evaluated due to low quality or large artifacts were left out of the analysis. Missing data were also left out of the analysis.

### Acquisition of clinical parameters

Clinical data obtained by experienced oral and maxillofacial surgeons from the University Hospital Leuven was retrospectively extracted from the patients’ medical files. Cause of injury, nerve involved, side of nerve involved, presence of pain, NST score, and clinical Sunderland class were extracted and recorded in a spreadsheet for further analysis.

### Statistical analysis

Statistical analyses were performed using RStudio Team (2020) (RStudio: Integrated Development for R. RStudio).

The required sample size was calculated based on pilot experiments suggesting a minimum of 20 participants when assuming 95% power and an *α* of 0.05.

To compare demographic data (sex, age) between cases and controls, the chi-squared test for sex and independent samples *t*-test for age were used.

The reliability of measurements was calculated for the following imaging parameters: presence of injury on MRN, nerve signal intensity (SI), nerve thickness, and MRN Sunderland classification score. Kappa coefficient and intraclass correlation coefficient were used. The interpretation of the Kappa coefficient value was as follows: < 0.00 poor agreement, 0.00 to 0.20 slight agreement, 0.21 to 0.40 fair agreement, 0.41 to 0.60 moderate agreement, 0.61 to 0.80 substantial agreement, and 0.81 to 1.00 almost perfect agreement [15]. For the intraclass correlation coefficient, the following interpretation was used: < 0.5 poor agreement, 0.5 to < 0.75 moderate agreement, 0.75 to < 0.9 good agreement, 0.9 to 1.0 excellent agreement [16].

Contingency tables were created to compare the presence of an injury as identified both clinically and through MRN. Within different data subgroups, the following statistical measures were calculated: sensitivity, specificity, positive predictive value (PPV), negative predictive value (NPV), positive likelihood ratio, and negative likelihood ratio.

The different subgroups of data were as follows:

- All data;
- Patients diagnosed with
  - PTN of the LN,
  - PTN of the IAN,
  - PTN and a low clinical Sunderland class,
  - PTN and a high clinical Sunderland class,
  - PTN of the LN and a low clinical Sunderland class,
  - PTN of the LN and a high clinical Sunderland class,
  - PTN of the IAN and a low clinical Sunderland class,
  - PTN of the IAN and a high clinical Sunderland class.

Classes I, II, and III were considered high. Classes IV and V were considered low.

To measure differences in the mean values of certain imaging parameters (aSNR, aNMCNR, and nerve thickness) between healthy and injured nerves, independent sample *t*-tests were used.

Correlation was determined using the Spearman correlation coefficient and predictive statistics used logistic regression with receiver operating characteristic analysis.

## Results

### Patient population

**Fig. 2:**
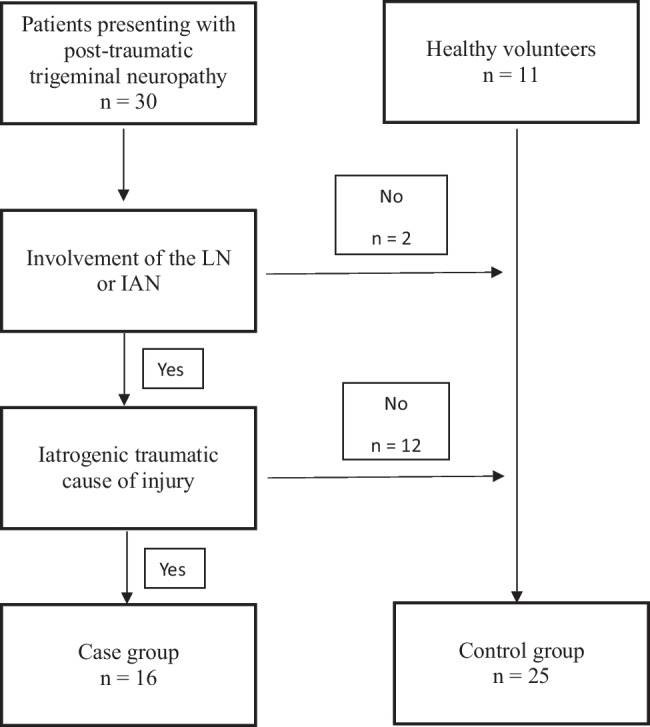
Study flowchart

All 41 participants were included in the final analysis. Sixteen patients were included in the case series. The other 14 patients were excluded due to neither the IAN nor LN being clinically suspected of being involved or no iatrogenic traumatic cause (Fig. 2). These patients were included in the control group, together with the 11 healthy volunteers (total *n* = 25).

The cases had a total of 18 injuries: 9 to the LN and 9 to the IAN. One patient had injuries to both lingual nerves and another to both inferior alveolar nerves. All other patients in the case series had an injury to a single nerve.

The case series consisted of 10 females and 6 males, and the control group of 16 females and 9 males. There was no significant difference in sex between cases and controls (*p* = 0.92).

Age in the case series varied between 16 and 62 years, with a mean age of 40 years. In the control group, age varied between 13 and 83 years, with a mean age of 51 years. The difference in mean age between both groups was not significant (*p* = 0.66).

Clinical data could be extracted from the medical files of all 16 patients in the case series. Neuropathic pain was present in nine patients. The others experienced neurosensory disturbances without them being described as painful.

**Fig. 3:**
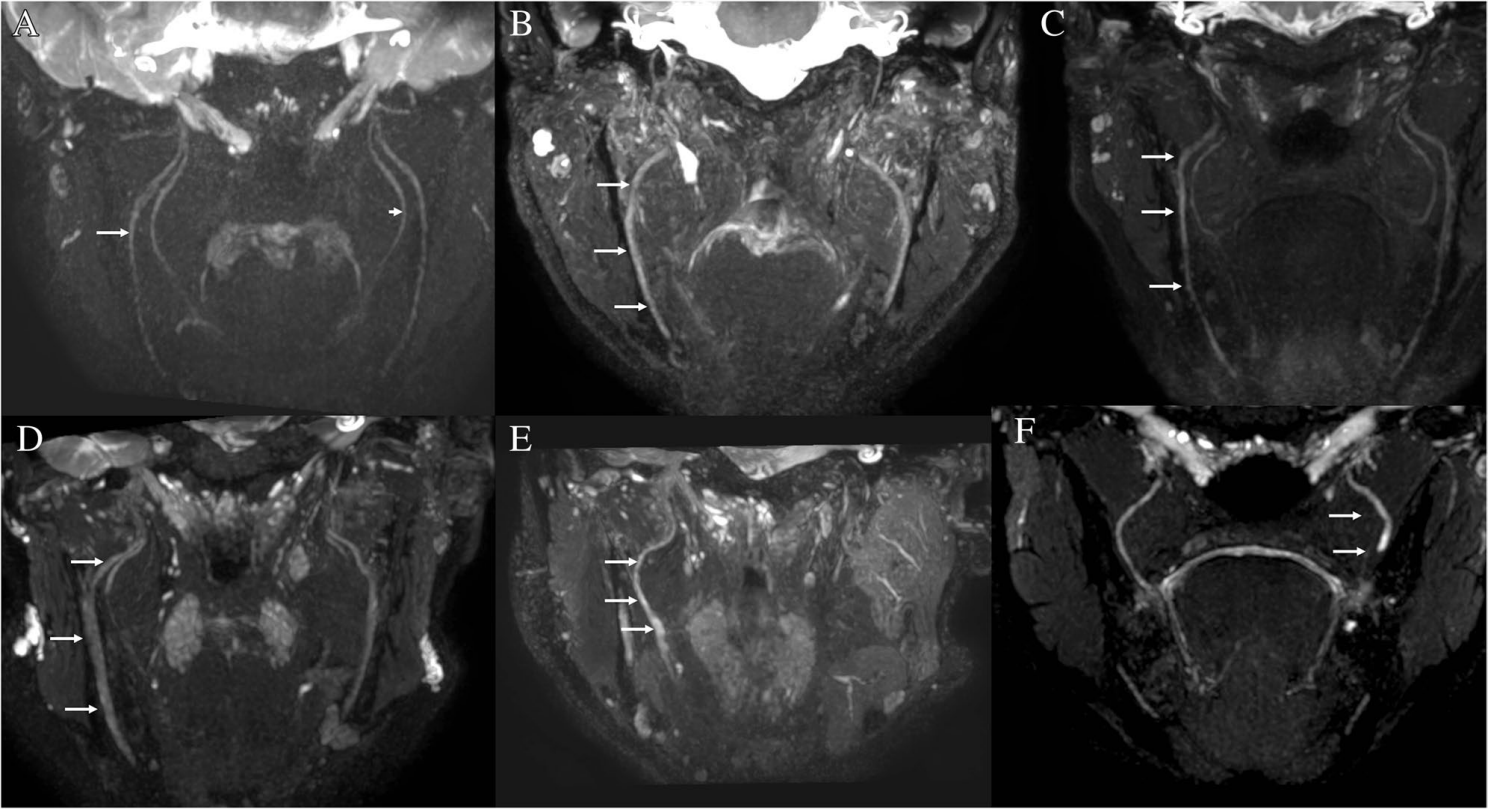
Illustrative case studies demonstrating varying degrees of nerve injury. Coronal plane 3D CRANI images. **A** Bilateral normal inferior alveolar nerve (IAN). **B** Grade I degree of right-sided IAN injury. Homogeneously increased T2 signal with no change in caliber. **C** Grade II degree of right-sided IAN injury. Homogeneously increased T2 signal and mild nerve thickening. **D** Grade III degree of right-sided IAN injury. Homogeneously increased T2 signal for nerve and moderate to marked nerve thickening, perineural fibrosis. **E** Grade IV injury of right IAN injury. Heterogeneously increased T2 signal and focal enlargement in otherwise continuous nerve. **F** Left, an end-bulb neuroma and transection of the left lingual nerve compatible with a class V injury

Clinical Sunderland classifications based on NST included eight class I, two class II, two class III, two class IV, three class V, and one undetermined injury (examples given in Fig. 3).

Iatrogenic causes of trauma were implant placement (*n* = 2), tooth extraction (*n* = 8), xanthoma curettage (*n* = 1), bilateral sagittal split osteotomy (BSSO; *n* = 1), BSSO + genioplasty (*n* = 1), open reduction internal fixation (ORIF; *n* = 1), and iatrogenic undefined (*n* = 2).

### Reliability of measurement

Inter-rater agreement for injury detected on MRN, nerve thickness, nerve signal intensity (SI nerve), and MRN Sunderland classification score was substantial, moderate, good, and moderate, respectively (Table 2).

**Table 2:** Reliability

Intra-rater agreement observer one	
Injury detected on MRN	0.51
Nerve thickness	0.55
Nerve signal intensity	0.9
MRN Sunderland classification	0.48
Intra-rater agreement observer two	
Injury detected on MRN	0.82
Nerve thickness	0.78
Nerve signal intensity	0.9
MRN Sunderland classification	0.63
Inter-rater agreement	
Injury detected on MRN	0.64
Nerve thickness	0.63
Nerve signal intensity	0.88
MRN Sunderland classification	0.55

*LN* lingual nerve, *IAN* inferior alveolar nerve

Intra-rater agreement for observer one for injury detected on MRN, nerve thickness, SI nerve, and MRN Sunderland classification score was moderate, moderate, excellent, and moderate, respectively (Table 2). For observer two, they were almost perfect, good, good, and substantial, respectively.

### Diagnostic accuracy

Overall, compared to NST, MRN had a sensitivity (true-positive rate) of 38.2% and specificity (true-negative rate) of 93.5% (Table 3). Positive likelihood ratio, negative likelihood ratio, positive predictive value, and negative predictive value were 5.9, 0.66, 46, and 91.3, respectively.

**Table 3:** Accuracy measures. Comparing the accuracy measures of detecting nerve injuries using MRN versus clinical neurosensory testing

	Sensitivity (%)	Specificity (%)	LR +	LR −	PPV	NPV
MRN overall	38.2	93.5	5.9	0.66	46	91.3
MRN low-grade injury	19.1	93.5	2.96	0.86	20.9	92.8
MRN high-grade injury	83.3	93.5	12.89	0.18	37	99.2
MRN LN injury	48.6	96.5	13.95	0.53	66.7	92.9
MRN IAN injury	28.2	90.7	3.02	0.79	30.6	89.7

*LR* + positive likelihood ratio, *LR − *negative likelihood ratio, *PPV* positive predictive value, *NPV* negative predictive value, *MRN* MR neurography, *IAN* inferior alveolar nerve, *LN* lingual nerve

When differentiated by clinical Sunderland class, both groups had a specificity of 93.5%. Sensitivity differed between both, with the low clinical Sunderland class group having a sensitivity of 19.1% and the high clinical Sunderland class having a sensitivity of 83.3%. Positive likelihood ratios in the low and high clinical Sunderland class groups were 2.96 and 12.89, respectively. The same tendency was seen when differentiated for both nerves. For the LN, the global sensitivity, specificity, and positive likelihood ratio were 48.6%, 96.5%, and 13.95. Differentiated according to clinical Sunderland class, specificity remained the same in both groups but sensitivity differed. Sensitivity and the positive likelihood ratio in the low clinical Sunderland class group were zero because of the absence of any true-positive results. In the high clinical Sunderland class group, sensitivity was 81.8%, with a positive likelihood ratio of 23.45.

The sensitivity, specificity, and positive likelihood ratio for the IAN group were 28.2%, 90.7%, and 3.02. Specificity remained the same when differentiated by clinical Sunderland class. In the presence of a low Sunderland class, the sensitivity and positive likelihood ratios were 25.7% and 2.76. For the higher classes, these values were 100% and 10.72, respectively.

### Correlation and prediction

There was a moderate, positive correlation between clinical and MRN Sunderland classification scores. The Spearman correlation coefficient based on the constructed contingency table was 0.53 (*p* = 0.005; Table 4).

**Table 4:** Contingency table indicating correlation between MRN and clinical injury severity

	MRN					
Clinical	I	II	III	IV	V	VI
I	0	0	0	0	0	0
II	0	1	1	0	0	0
III	2	1	1	1	0	0
IV	0	0	0	0	6	0
V	0	3	0	0	9	1
VI	0	0	0	0	3	0

*MRN* MR neurography. VI represents the answer “indeterminate”

The prediction model using aSNR, aNMCNR, and nerve thickness to predict the presence of injury had an area under the receiver operating characteristic curve of 0.78 (*p* =  < 0.05), with an *F*-score of 0.19 and an accuracy rate of 0.89. The permutation feature importance test showed the following levels of importance for the different variables: 408.03 for aNMCNR (*p* =  < 0.05), 293.33 for aSNR (*p* =  < 0.05), and 28.50 for nerve thickness (*p* =  < 0.05). Additional receiver operating characteristic analyses of aSNR in combination with nerve thickness and aNMCNR in combination with nerve thickness were performed due to the multicollinearity between aSNR and aNMCNR. The area under the receiver operating characteristic curve, *F*-score, and accuracy rate were 0.73, 0.01, and 0.89, respectively (*p* =  < 0.05), for the model using aSNR and 0.77, 0.96, and 0.92 (*p* =  < 0.05) for the model using aNMCNR.

### Descriptive statistics

Differences in aSNR, aNMCR, and nerve thickness between healthy and injured nerves are shown in Table 5. A significant difference in mean nerve thickness was found for the overall dataset but not for both nerves separately. For aSNR and aNMCNR, a significant difference was found for the overall dataset and both nerves separately.

**Table 5:** Nerve diameter and apparent signal intensity measurements

	Overall			LN			IAN		
Variable	Injured	Healthy	*p*-value	Injured	Healthy	*p*-value	Injured	Healthy	*p*-value
Nerve thickness (mm)									
Mean (SD)	1.77 (0.94)	1.62 (1.07)	.033	1.29 (0.84)	1.38 (0.55)	.8	1.29 (0.84)	1.38 (0.55)	.8
Median (IQR)	1.70 (1.37, 2.21)	1.59 (1.25, 1.95)		1.48 (0.81, 1.73)	1.42 (1.17, 1.74)		1.48 (0.81, 1.73)	1.42 (1.17, 1.74)	
Range	0.00, 4.09	0.00, 21.49		0.00, 2.68	0.00, 3.20		0.00, 2.68	0.00, 3.20	
aSNR									
Mean (SD)	180 (138)	119 (84)	< .001	207 (176)	109 (75)	.002	207 (176)	109 (75)	.002
Median (IQR)	149 (97, 251)	103 (59, 164)		185 (87, 363)	97 (49, 157)		185 (87, 363)	97 (49, 157)	
Range	0, 561	0, 450		0, 561	0, 370		0, 561	0, 370	
aNMCNR									
Mean (SD)	114 (114)	68 (62)	< .001	136 (151)	59 (55)	.001	136 (151)	59 (55)	.001
Median (IQR)	86 (53, 147)	60 (31, 97)		101 (52, 252)	52 (27, 87)		101 (52, 252)	52 (27, 87)	
Range	− 66, 455	− 129, 310		− 66, 455	− 129, 236		− 66, 455	− 129, 236	

*aNMCNR* apparent nerve-muscle contrast-to-noise ratio, *aSNR* apparent signal-to-noise ratio, *IAN* inferior alveolar nerve, *IQR* interquartile range, *LN* lingual nerve, *SD* standard deviation

## Discussion

In current practice, the diagnosis and stratification of injuries to the LN and IAN in patients with PTN are based on NST, but this approach has limitations. MRN, a nerve-selective MRI technique, has shown potential as a more standardized and reliable tool in detecting and stratifying these lesions and providing additional information about location and other anatomical specifications (Fig. 3). The latter would be very useful in surgical planning. Therefore, our goal was to determine whether MRN is an accurate tool in diagnosing these injuries.

Overall, MRN had a good specificity of 93.5% but a rather low sensitivity of 38.2%, which accounts for a high rate of false-negative results. Differentiating by the degree of injury using the clinical Sunderland classification system, we found higher sensitivity in the presence of a higher classification score. Lingual nerve injuries with a clinically high degree of injury had a sensitivity of 81.8% with a positive likelihood ratio of 23.45. For inferior alveolar injuries, the values were 100% and 10.72, respectively.

Compared to conventional MRI, for which a previous study had calculated a sensitivity of 0.18 [17], MRN performs much better in detecting nerve injuries. This was expected knowing the specific characteristics of MRN [12].

A high sensitivity (i.e., low false-negative rate) is vital for detecting a certain condition or disease, such as the presence of a peripheral nerve injury. The sensitivity of MRN in the presence of a lower clinical Sunderland class was not great, but this would be of lesser importance when considering the specific use of MRN in practice. In clinical practice, MRN would not be offered to every patient presenting with PTN. Logically, because of its additional benefits in providing information about the location and anatomical specifications of the injury, MRN would be of greater usefulness to patients with a higher degree of damage who are eligible for surgery as a possible treatment option.

For a clinician, understanding the context in which MRN can contribute to medical decision-making is important. If a low degree of damage clinically is suspected, the change in accurately visualizing damage via MRN is rather low due to its high false-negative rate in this context. Therefore, a good clinical diagnosis is necessary before making the decision to use MRN for further investigation and visualization of damage.

Stratifying the degree of injury using MRN positively correlated with clinical stratification using NST. These results are in accordance with a previous study [8] in which a positive correlation was found between Sunderland classes based on MRN and NST. That study also compared the degree of injury on MRN with surgical findings, finding a positive correlation [8].

This study also showed the possible application of aSNR, aNMCNR, and nerve thickness as quantitative imaging markers for peripheral nerve injuries. Injured nerves had a significantly higher mean value for these parameters. Previous studies already confirmed increased signal intensities correlating with nerve injury [8–11]. However, to date, we lack histological correlation with MRN findings, which would be interesting to investigate further. In addition, our prediction model showed the ability of these variables to accurately predict whether an injury is present. Because of the multicollinearity between aSNR and aNMCNR, one of these in combination with nerve thickness should be sufficient. Accuracy did not differ in separate models (aSNR in combination with nerve thickness and aNMCNR in combination with nerve thickness), but feature importance and regression coefficient analysis showed a preference for aNMCNR over aSNR. This was confirmed by comparing the areas under both models’ receiver operating characteristic curves.

This study had some limitations. Excluded patients who were diagnosed with neuropathy in the orofacial region were included as controls. It is not clinically possible to exclude the presence of damage to the LN and IAN. Therefore, there is the possibility that individual nerves in these patients were falsely classified as false-positives.

Also, we used NST as our reference test knowing that NST itself is not perfect in detecting peripheral nerve injuries. This decision was made because we could not use surgical findings for ethical reasons.

Finally, both observers were junior researchers who, although they received a short course and calibration session on MRN, were not senior radiologists with a vast experience in MRN evaluation. This could enhance the number of false results and could be a reason why MRN was not successful in detecting injuries in the presence of a low clinical Sunderland class.

In future research, we suggest using surgical findings as the reference test if it is ethically possible (e.g., using retrospective surgical data) and assigning experienced radiologists as observers.

## Abbreviations

aNMCNR: Apparent nerve-muscle contrast-to-noise ratio
aSNR: Apparent signal-to-noise ratio
IAN: Inferior alveolar nerve
LN: Lingual nerve
MRN: MR neurography
NST: Neurosensory testing
PTN: Post-traumatic trigeminal neuropathy
TN: Trigeminal nerve

## Declarations

### Guarantor

The scientific guarantor of this publication is Fréderic Van der Cruyssen.

### Statistics and biometry

Wim Coucke kindly provided statistical advice for this manuscript.

### Study subjects or cohorts overlap

Some study subjects or cohorts have been previously reported in Casselman J, Van der Cruyssen F, Vanhove F, et al 3D CRANI, a novel MR neurography sequence, can reliable visualise the extraforaminal cranial and occipital nerves. Eur Radiol. Published online November 26, 2022.

The healthy subjects (“controls”) in the current case–control study were also reported in this previous publication.

### Methodology

• prospective

• diagnostic case–control study

• performed at one institution
